# Central circuits in control of energy expenditure: connecting energy and temperature homeostasis

**DOI:** 10.1007/s00360-026-01688-7

**Published:** 2026-07-27

**Authors:** Clint Kinney, Laura Kaiser, Christopher D. Morrison, Hans-Rudolf Berthoud, Heike Münzberg

**Affiliations:** 1 Neurobiology of Nutrition and Metabolism Department, Pennington Biomedical Research Center (PBRC), LSU Louisiana State University System System, 6400 Perkins Rd, Baton Rouge, LA 70808, USA

**Keywords:** Arcuate nucleus, Paraventricular hypothalamus, Preoptic area, Dorsomedial hypothalamus, Energy sensing, Temperature sensing

## Abstract

Energy expenditure (EE) is the dynamic physiological process that balances energy intake to maintain body weight and body temperature. Environmental changes induce EE adaptations that limit fluctuations in body weight or body temperature by balancing between body temperature defense and energy conservation. The central nervous system (CNS) orchestrates these adaptations by integrating multiple external and internal cues to coordinate thermogenesis together with other behavioral and metabolic processes. Key hypothalamic and brainstem nuclei form a distributed network that modulates autonomic output to peripheral organs, including sympathetic tone to brown and white adipose tissues. Here, we review known neural circuits and neurotransmitter systems, their integration with sensory pathways and functionally opposing output discrepancies that reveal knowledge gaps how the CNS switches between body temperature defense and energy preservation.

## Introduction

Energy and body temperature homeostasis require coordination of both energy intake and expenditure, as well as heat production and heat loss. Beyond food intake, the central mechanisms that govern energy expenditure (EE) are fundamental for body weight, body temperature and metabolic health. EE includes basal metabolic rate, physical activity (including shivering) and adaptive (non-shivering) thermogenesis via brown adipose tissue (BAT). This review focuses on central circuits involved in adaptive thermogenesis (for shivering and vasomotion see (Morrison and Nakamura 2019; Madden and Morrison 2019).

The CNS receives environmental and internal signals that reflect thermal, visceral and humoral inputs (e.g. cutaneous and spinal thermoreceptors, vagal afferents and hormones) that are relayed into thermogenic and behavioral responses. The involved neuronal networks link several hypothalamic nuclei with the brainstem and autonomic outflow to thermoeffectors such as BAT. A variety of modern tools including conditional genetic targeting, opto/chemogenetics, projection-specific manipulations, fiber photometry and single-cell/spatial transcriptomics, have revealed substantial cellular heterogeneity and neurochemical diversity in brain nuclei that underscore the complexity of how these circuits fine-tune metabolic output. Yet, despite substantial progress we are still lacking a mechanistic framework to explain how the brain integrates exteroceptive and interoceptive signals to prioritize EE induction versus suppression. This review highlights current understanding, unresolved questions and outlines emerging directions toward closing these gaps.

## Hypothalamic regulation of EE

### Arcuate nucleus (ARC)

The ARC integrates humoral signals (e.g. leptin, insulin, ghrelin) through access to the median eminence (ME) and tanycytes (Faouzi et al. 2007; Langlet et al. 2013). Within the ARC, proopiomelanocortin (POMC) neurons promote negative energy balance with increased EE via melanocortin-4 receptor (MC4R) signaling in downstream targets, whereas agouti-related peptide (AGRP) neurons antagonize MC4R signaling to suppress EE (Schwartz et al. 2000) (Fig. 1A). Fasting increases AGRP activity and reduces POMC activity, while refeeding reverses these changes (Chen et al. 2015). Similarly, chemogenetic activation of AGRP neurons recapitulates fasting-associated EE reduction (Krashes et al. 2011).

Some POMC neurons are multi-synaptically connected to sympathetic BAT inputs (BAT-related neurons). BAT-related neurons can be visualized with pseudorabies virus injected into BAT which is retrogradely and transsynaptically transported from sympathetic nerve terminals to CNS neurons that are related to the modulation of sympathetic BAT inputs (Oldfield et al. 2002; Cano et al. 2003). These BAT-related POMC neurons are found in the rostral ARC and project to the spinal cord (Elias et al. 1998; Oldfield et al. 2002) where MC4R is expressed in pre-ganglionic sympathetic neurons; and genetic *Mc4r* deletion from pre-ganglionic sympathetic neurons significantly reduced EE (Berglund et al. 2014). However, selective activation of POMC neurons with opto/chemogenetics has not been validated to induce EE.

Beyond the energy state dependent effects of MC4R signaling (Garfield et al. 2016), ARC neurons participate in temperature-dependent adaptations. AGRP neurons are strongly activated by cold exposure to promote cold-induced feeding without affecting EE (Deem et al. 2020). POMC neurons show intrinsic warm-sensitivity to promote warm-temperature induced satiety (Jeong et al. 2018; Kaiser et al. 2025). Thus, ambient temperature-induced neuronal activity in ARC neurons (activated POMC = FI↓/EE↑; activated AGRP = FI↑/EE↓) is consistent with expected warm-suppressed and cold-induced feeding responses, but inconsistent with the expected warm-suppressed and cold-induced EE adaptations. Together, these findings reveal that ARC neurons encode energy state but do not determine thermogenic output during thermal stress. This implies that thermoregulatory circuits are hierarchically dominant over ARC circuits with regards to EE and this important circuit logic remains unresolved.

### Paraventricular hypothalamus (PVH)

The PVH integrates hypothalamic and brainstem inputs and relays them via endocrine, autonomic (sympathetic and parasympathetic) and behavioral outputs (Fig. 1A). The PVH is also the main location of endocrine TRH circuits that stimulate thyroid release via pituitary and thyroid gland (for review see (Fekete and Lechan 2014)). PVH MC4R signaling has been linked to EE control, because A) these neurons are BAT-related (Voss-Andreae et al. 2007), B) intra-PVH infusion of MC4R agonists increase BAT temperature and thermogenic gene expression (Voss-Andreae et al. 2007; Song et al. 2008; Monge-Roffarello et al. 2014) and C) PVH MC4R neurons show cold-induced activation (cFos-induction) while warm-exposure suppressed cFos induction (Cano et al. 2003; Kaiser et al. 2025), indicating that PVH MC4R neurons mediate cold-induced EE but not cold-induced FI. In contrast, select *Mc4r* re-expression in PVH neurons of *Mc4r*-null mice rescues hyperphagia without normalizing low EE (Balthasar et al. 2005; Shah et al. 2014) and chemogenic PVH^*Mc4r*^ activation reduced feeding but does not alter EE (Garfield et al. 2015), indicating that PVH MC4R neurons exclusively mediates feeding but not EE adaptations.

PVH oxytocin neurons innervate sympathetic premotor neurons in the raphe pallidus (RPa) and spinal preganglionic neurons (Swanson and Sawchenko 1980). Their chemogenetic activation increases sympathetic tone to BAT, BAT temperature and EE (Sutton et al. 2014; Fukushima et al. 2022). Overall chemogenetic PVH stimulation (including oxytocin and MC4R neurons), robustly increases EE and BAT temperature (Sutton et al. 2014), while others showed PVH NMDA stimulation in anesthetized rats either had no effect or blocked cold-induced BAT thermogenesis (Madden and Morrison 2009). Moreover, neuropeptide Y (NPY) signaling in the PVH (possibly originating from ARC-AgRP neurons) suppressed BAT thermogenesis via multisynaptic inhibition of RPa premotor neurons (Nakamura et al. 2017).

These opposing results indicate that the PVN contains functionally antagonistic EE modulating neurons. The identification of their molecular signature and projection logic is essential for understanding how the PVH is able to switch between energy-expending and -conserving states.

### Preoptic area (POA)

The POA is a key thermoregulatory site that receives cutaneous warm/cold signals via the lateral parabrachial nucleus (Nakamura and Morrison 2010, Yahiro 2023). Warm-sensitive POA neurons increase firing with rising ambient temperature (Boulant and Dean 1986), suppress BAT thermogenesis, and promote heat dissipation (Nakamura and Morrison 2007) (Fig. 1B).

Recent studies identified a largely homogenous population of warm-sensing, glutamatergic POA neurons that potently suppress EE, drive body cooling and exhibit intrinsic temperature-sensitivity and marker genes include leptin receptor (*Lepr*), warm temperature sensor Trpm2 and light sensor Opn5 (Yu et al. 2016; Song et al. 2016; Abbott and Saper 2017; Moffitt et al. 2018; Takahashi et al. 2020; Zhang et al. 2020a). While earlier circuit models (mostly based on experimental work in rats) emphasized a GABAergic identity of these POA neurons to inhibit downstream cold-activated neurons in the DMH and RPa (Morrison and Nakamura 2019), genetic studies in mice showed that the use of Gad1/Gad2 is unreliable for distinguishing inhibitory POA neurons (Moffitt et al. 2018), thus downstream inhibition likely involves interposed inhibitory interneurons and/or spinal projecting neurons within the RPa (Fig. 1B).

The POA is well positioned to integrate many signals such as sex hormones (Zhang et al. 2020b), inflammation/fever-inducing signals (Lazarus et al. 2007; Machado et al. 2025) and daylight-induced signals (Fetsch et al. 2006). POA neurons show robust induction of pS6, as a proxy for neuronal activation, in response to cold exposure, but remain unchanged in response to fasting conditions (Yamaguchi et al. 2023). Nevertheless, POA leptin signaling significantly contributes to energy state-dependent metabolic changes such as fasting and overfeeding, e.g. POA^*Lepr*^ ablation prevents fasting-suppressed EE (Yu et al. 2018). Because fasting does not affect POA activation and hypometabolism is attributed to ARC-AgRP neurons, integration likely occurs downstream of POA temperature-sensing and ARC energy sensing. This integration may be relevant for other hypometabolic states such as torpor behavior (for further literature see (Ambler et al. 2021; Cerri 2024; Ohba and Yamaguchi 2025)), which can be induced by combining fasting and cold-exposure specifically in female mice (Oelkrug et al. 2011). Synthetic activation of warm-sensing POA neurons (*Lepr*, *Opn5*, *Trpm2* expressing neurons) mimics EE suppression observed in torpor (Yu et al. 2016; Song et al. 2016; Zhang et al. 2020a), even though full torpor features (e.g. REM sleep, unresponsive to touch (Huang et al. 2021)) are not replicated (Yu et al. 2016).

Cold-activated POA neurons are less characterized, but are also glutamatergic, and are clearly distinct from warm-activated neurons and may drive opposing defensive behaviors compared with warm-sensing POA neurons (Yu et al. 2016; Song et al. 2016; Abbott and Saper 2017; Yang et al. 2021). While the POA is well accepted as the brain’s primary thermal control site, without a select marker for homogenous cold-sensing POA neurons the functional distinction of opposing glutamatergic POA populations is difficult and stimulation of heterogenous POA populations likely explains inconsistent patterns of EE vs. body temperature. A marker for homogenous cold-activated POA neurons would be an important step forward to better understand the mechanistic interplay of warm- vs. cold-activated neurons and how these outputs are weighted to enable body temperature defense vs. hypometabolism.

### Dorsomedial hypothalamus (DMH)

The DMH integrates thermoregulatory, circadian, sleep and stress-related inputs to translate into EE adaptations. Early studies by DiMicco and colleagues demonstrated that chemical stimulation or inhibitory input blockade of DMH neurons promotes body temperature increases (Zaretskaia et al. 2002, 2003). Subsequent work established the POA → DMH → RPa axis as a key pathway for cold- and fever-induced BAT activation in anesthetized rats (Nakamura and Morrison 2007).

The role of the DMH in whole body EE, beyond thermoregulation, became evident when BAT-related DMH neurons were found to express *Lepr* and *Mc4r* (Voss-Andreae et al. 2007; Zhang et al. 2011). Leptin microinfusion into the DMH normalizes hypothermia in leptin-deficient *ob/ob* mice, and chemogenetic DMH^*Lepr*^ activation increases EE and BAT thermogenesis without affecting food intake (Rezai-Zadeh et al. 2014). Conversely, DMH-specific deletion of *Lepr* or *Mc4r* increases body weight primarily by reducing EE. Notably, DMH^*Mc4r*^ deletion does not impair cold-induced thermogenesis, whereas DMH^*Lepr*^ deletion does, indicating distinct roles for each receptor (Rezai-Zadeh et al. 2014; Chen et al. 2017). DMH^*Lepr*^ neurons also shape circadian rhythms of EE, locomotion and feeding. Blocking neurotransmission in DMH^*Lepr*^ neurons advances diurnal rhythms into the light cycle and blunts rhythmic EE and locomotor amplitude (Faber et al. 2021). It remains unclear whether leptin action drives this shift through neuronal activation, transcriptional changes, or other mechanisms.

Other hypometabolic states such as torpor and hibernation are also following circadian or circannual rhythms (Körtner and Geiser 2000). Such rhythms could derive from an interaction of the suprachiasmatic nucleus (SCN) and DMH, as suggested for food anticipatory behavior in mice (Acosta-Galvan et al. 2011) but may also require gating by permissive factors including hormones, sympathetic tone, and NREM sleep (Swoap et al. 2006; Huang et al. 2021) to prevent hypometabolism at inappropriate times (e.g. running from a predator).

In thermoregulatory circuit models, glutamatergic BAT-related DMH neurons mediate fever and cold-induced thermogenesis via RPa projections (Machado et al. 2018). Those RPa-projecting DMH neurons co-express markers (*Mc4r*, *Ntrk2* and *Bsr3*) that are also linked to EE induction (Song et al. 2008; Houtz et al. 2021; Piñol et al. 2018). However, chemogenetic activation of both inhibitory (Vgat expressing) and stimulatory (Vglut2 expressing) DMH neurons robustly induce EE, but only DMH^*Vglut2*^ neurons innervate the RPa, while DMH^*Vgat*^ neurons innervate the medial ARC. Only DMH^*Lepr*^ activation, with mixed inhibitory and stimulatory neurons, effectively induces BAT-thermogenesis and promotes body fat loss (Francois et al. 2025), suggesting that RPa projecting DMH neurons may be necessary but not sufficient to fully activate BAT thermogenesis.

Evidence for EE-suppressing DMH neurons includes stimulation of cholinergic DMH neurons (Jeong et al. 2015), stimulation of POA^*Pacap/Bdnf*^ or POA^Qrfp^ projections to the DMH (Tan et al. 2016; Takahashi et al. 2020), or neuronal stimulation of POA^EP3R^ that consist of DMH projecting POA^*Pacap/Bdnf*^ and POA^Qrfp^ neurons (Machado et al. 2025), which all reduce body temperature when stimulated. Yet, only POA^*Qrfp*^ → DMH fiber activation replicated the strong suppression of body temperature observed following POA^*Lepr*^ stimulation and able to suppress cold-induced thermogenesis (Yu et al. 2016; Takahashi et al. 2020; Zhang et al. 2020a; Ambroziak et al. 2025) and further supports the existence of EE-suppressing neurons in the DMH that are able to shift the organism from default body temperature defense (cold-induced thermogenesis) to energy preservation (fasting-induced hypometabolism, torpor, hibernation). Other effective approaches identified EE-suppressing DMH neurons by targeting torpor-induced neurons, achieved in fasted female mice with acute exposure to cold (Oelkrug et al. 2011; Ambler et al. 2022; Yamaguchi et al. 2023). Similar to hibernation, torpor results in an extended temperature range where body temperature is no longer defended unless ambient temperature falls under a lower threshold (Buck and Barnes 2000).

POA^*Ptrg3*^ and POA^*Brs3*^ neurons both show opposing bidirectional effects on body temperature and both innervate the DMH (Piñol et al. 2021; Nakamura et al. 2022). As such, either inhibition or stimulation of these DMH projections, respectively, increase body temperature. For inhibition of POA^*Ptrg3*^ → DMH projections induction of BAT thermogenesis was further confirmed by demonstrating increased sympathetic activation, increased BAT temperature, and increased CO_2_ production. Both populations may provide mixed GABA and glutamatergic inputs to the DMH and mixed warm- and cold-sensing projections which complicate interpretation for DMH circuit logics. However, together these data suggest that the POA → DMH axis is capable to mediate suppression, induction and maintenance and induction of body temperature and EE via distinct neuronal populations.

Anatomically, BAT-related neurons cluster in the dorsal DMH and dorsal hypothalamic area where most glutamatergic neurons are found. In contrast, GABAergic neurons are broadly distributed throughout the DMH (Francois et al. 2025). The compact DMH (cDMH) is notable for inducible NPY expression that impacts EE and BAT thermogenesis through hypothalamic projections, especially to the PVH (Chao et al. 2011; Lee et al. 2013).

Overall, the DMH emerges as a key integrator that can arbitrate between conflicting inputs and demands (fasting vs. cold) to prioritize body-temperature defense or allow energy-saving hypometabolism. Yet, the mechanistic circuit logic remains unknown and represents a fundamental open question in thermal biology.

## Brainstem and autonomic control of thermogenesis

### Raphe pallidus and spinal cord

The RPa (and surrounding areas) serves as the principal premotor center for sympathetic outflow to BAT and other sympathetic functions (e.g. skeletal muscle shivering, cutaneous vasculature) (Nakamura et al. 2005). They are BAT-related neurons that are activated by cold-exposure (Cano et al. 2003), and activation of RPa neurons induces, while inhibition suppresses BAT thermogenesis measured by changes in BAT temperature and CO_2_ production (Madden and Morrison 2003; Nakamura et al. 2005) which suggests their putative role as EE-inducing RPa neurons. The RPa is also well connected with other EE controlling hypothalamic sites such as PVH, POA and DMH but the interplay of inhibitory and excitatory inputs remains incomplete.

Earlier studies suggested a direct inhibitory input from warm-sensing POA neurons, while recent data suggest a major glutamatergic input from warm-sensing POA neurons. Thus, suppression of putative EE-inducing RPa neurons by glutamatergic warm-sensing neurons would require transductions via local inhibitory interneurons or spinal projecting inhibitory RPa neurons (Stornetta et al. 2005; Yu et al. 2016; Münzberg et al. 2020; Machado and Saper 2022) (Fig. 1B).

Excitatory projections from the POA and DMH converge in the RPa and their opposing effect on EE suggests that their RPa projections target distinct RPa neurons. Projection specific retrograde tracing and inhibition of glutamatergic DMH → RPa projecting neurons demonstrated their anatomic connectivity with excitatory RPa neurons, and their stimulation increased EE while inhibition caused suppression of body temperature and blunted stress-induced thermogenesis (Machado et al. 2018). However, compared to the strong EE and body temperature suppression observed by stimulation of warm-sensing POA neurons or torpor inducing DMH neurons, these glutamatergic DMH → RPa inputs seem to account for only mild effects on EE and body temperature.

Within the spinal cord, pre-ganglionic neurons are contacted by a network of excitatory and inhibitory interneurons from the brainstem (Stornetta et al. 2005) as well as local spinal interneurons that may all provide local feedback loops. Currently, a systematic circuit mapping for GABAergic and glutamatergic monosynaptic inputs to the RPa or pre-ganglionic neurons as well as their functional impact on EE is missing and represents a key research gap to understand how these inputs are linked to BAT thermogenesis, EE and overall energy homeostasis.

Beyond the POA and DMH, the PVH provides additional regulatory control over the RPa and spinal circuits, adding complexity to EE modulation. Body temperature and body weight homeostasis both leverage EE modulation, and the PVH provides indirect and direct inputs to the RPa as well as direct inputs to the spinal cord (Swanson and Sawchenko 1980; Geerling et al. 2010; Sutton et al. 2014; Nakamura et al. 2017). Similarly, some ARC^*Pomc*^ neurons also directly innervate the spinal cord (Elias et al. 1998) and thus both could provide powerful effects on EE as they could circumvent thermoregulatory signals from the RPa. The logic of these circuits might help us to understand why fasted and cold-exposed mice can switch into a torpor instead of increasing EE to defend body temperature.

The PVH is also multisynaptically connected to the RPa allowing powerful suppression of BAT thermogenesis (Madden and Morrison 2009; Nakamura et al. 2017). Furthermore, RPa neurons innervate the dorsal vagal complex where the nucleus of the solitary tract (NTS) receives sensory para-sympathetic input specifically from the gastrointestinal (GI) tract, while the dorsal motor nucleus of the vagus (DMV) provides vagal motor outflow to the GI tract and mediates cold-induced gastric motility (Palkovits et al. 1986; Martínez et al. 2001). Therefore, the PVH → RPa → DMV may enable interactions of spinal and vagal functions to maintain energy and temperature homeostasis. The PVH seems to mediate both increased EE as well as suppression of EE, suggesting the existence of a powerful inhibitory axis that may be masked by stimulation of a larger PVH population. Resolving these conflicting findings is an important future task that may reveal the critical mechanisms that govern EE switches during competing states, such as fasting vs. cold exposure.

In summary, the RPa is a critical hub for sympathetic control of BAT thermogenesis, integrating excitatory and inhibitory inputs from hypothalamic and brainstem circuits. However, the precise organization of these pathways, especially the roles of inhibitory vs. excitatory inputs and outputs, remains unresolved and may be key to understanding how competing signals from POA, DMH, and PVH (e.g. cold-defensive vs. torpor) are prioritized.

## Peripheral targets, humoral factors and feedback loops

BAT thermogenesis depends on sympathetic innervation and β3-adrenergic activation. Yet, brown adipocytes alone may not suffice for efficient thermogenesis. For example, whole-body deletion of β3-adrenergic receptors (β3AR) completely abolishes the increase in EE induced by a β3-selective agonist. However, select re-expression of β3AR only in brown adipocytes is insufficient to restore β3AR agonist induced EE, whereas re-expression in both brown and white adipocytes restores this effect (Grujic et al. 1997). Similarly, sympathetic induction of lipolysis fuels BAT thermogenesis, but genetic ablation of lipolysis in brown adipocytes is dispensable for cold-induced thermogenesis and instead lipolysis in cardiomyocytes and white adipocytes is relevant for effective cold-induced thermogenesis (Schreiber et al. 2017; Shin et al. 2017).

In addition, sympathetic drive can modulate humoral factors, such as the requirement of a the sympathetic tone to reduce leptin levels (Commins et al. 2000; Swoap et al. 2006). Peripheral tissues release various humoral factors in insects as well as mammals (Droujinine and Perrimon 2016) and we have just begun to understand some of their impacts on interoceptive feedback and feedforward systems. Thyroid hormones are also an important humoral outflow axis that is required for basal metabolic rate in peripheral tissues (Mullur et al. 2014). Thyroid hormones may also affect sensory and central nerves, though such feedback loops within central EE circuit have been largely unexplored. The increased use of public data from transcriptomic studies may reveal more information for humoral receptor expression and other peripheral factors in central and sensory neurons in the future to unravel these important feedback axes.

## Conclusions

Central circuits for EE form an interconnected network that spans hypothalamic (ARC, PVH, POA, DMH), brainstem (RPa), and autonomic nodes. A unifying principle that emerges from recent work is that opposing thermogenic and energy-conserving responses require circuits that allow switches between cold-defense or energy conservation. How the brain implements this switch, e.g. which inputs are weighted, integrated or exert hierarchical control, remains a major unresolved question. A limitation in the field is the absence of molecular definitions that distinguish functionally opposing neurons within key nodes such as PVH, POA and DMH. In addition, current labeled-line models do not adequately explain circuit bias, masking and overriding phenomena repeatedly observed by synthetic stimulation of distinct populations, projections or specific context. Understanding how these principles operate is essential for predicting when the brain prioritizes body-temperature defense over hypometabolic energy saving.

Insights from these circuits can inform applied questions such as whether warm ambient temperature in anorexia enhances unwanted feeding suppression or whether this effect is outweighed by the beneficial EE suppression (Gutierrez et al. 2024). Defining the molecular logic and plasticity of EE circuits will be crucial for identifying environmental and physiological states that boost resilience versus vulnerability to obesity, anorexia, and metabolic disease and may reveal new therapeutic targets that can be leveraged to restore energy balance.

## Figures and Tables

**Fig. 1:**
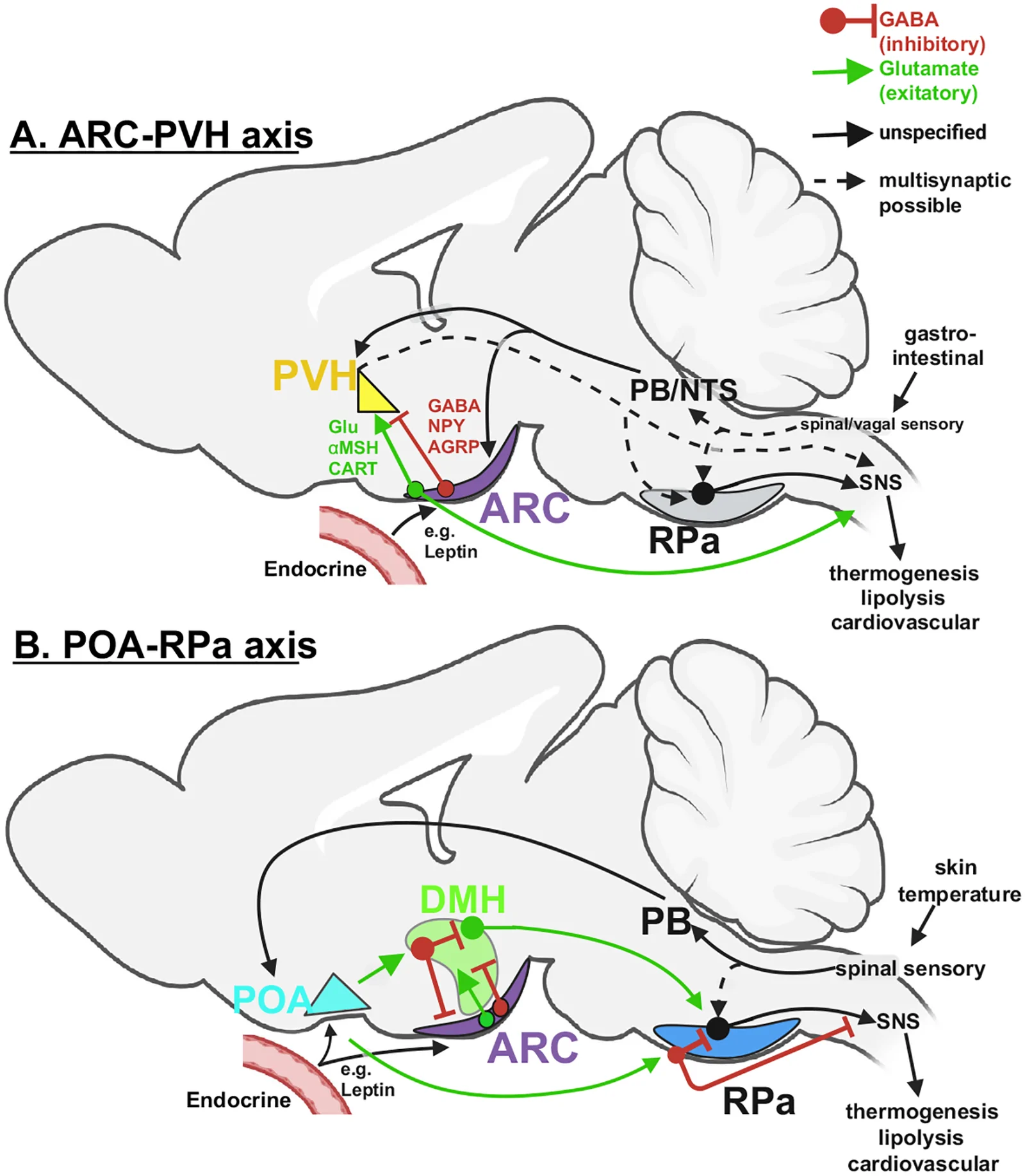
Central energy expenditure circuits. Schematic summary of major energy expenditure circuits that are most prominently associated with energy sensing and body weight homeostasis (**A**), compared to energy expenditure circuits that are most prominently associated with temperature sensing and body temperature homeostasis (**B**). These circuits both have several nodes where extensive integration or prioritization of temperature vs. energy sensing may occur. *ARC* arcuate nucleus, *PVH* paraventricular hypothalamus, *Glu* glutamate, *αMSH* alpha-melanocortin-stimulating-hormone, *CART* cocaine-and-amphetamine-responsive-transcript, *NPY* neuropeptide Y, *AGRP* agouti-related-peptide, *PB* parabrachial nucleus, *NTS* nucleus of the solitary tract, *RPa* raphe pallidus, *SNS* sympathetic nervous system, *POA* preoptic area, *DMH* dorso-medial hypothalamus

## Data Availability

No new data were created or analyzed in this study. Data sharing is not applicable to this article.
